# Supplementary material for: Fluorine-Nitrogen-Codoped Carbon Dots as Fluorescent Switch Probes for Selective Fe(III) and Ascorbic Acid Sensing in Living Cells
Source: Molecules. 2022 Sep 20;27(19):6158. doi: 10.3390/molecules27196158 (PMC9572543; doi:10.3390/molecules27196158)
Supplement: Supplementary file 1 [file molecules-27-06158-s001.zip › molecules-1931829-supplementary.pdf]

Supplementary information

Fluorine-Nitrogen-Codoped Carbon Dots as Fluorescent Switch Probes for Selective Fe(III) and Ascorbic Acid Sensing in Living Cells

Shuai Ye, Mingming Zhang, Jiaqing Guo, Xiantong Yu, Jun Song, Pengju Zeng, Junle Qu, Yue Chen * and Hao Li *

Shenzhen Key Laboratory of Photonics and Biophotonics, Key Laboratory of Optoelectronic Devices and Systems of Ministry of Education and Guangdong Province, College of Physics and Optoelectronic Engineering, Shenzhen University, Shenzhen 518060, China

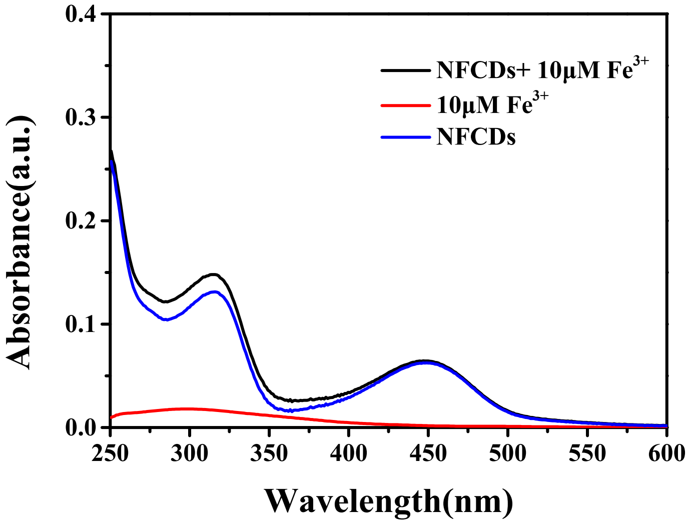

Figure S1. Absorption spectra of NFCDs (blue line), Fe^{3+} (red line), and NFCDs quenched by Fe^{3+} (black line).

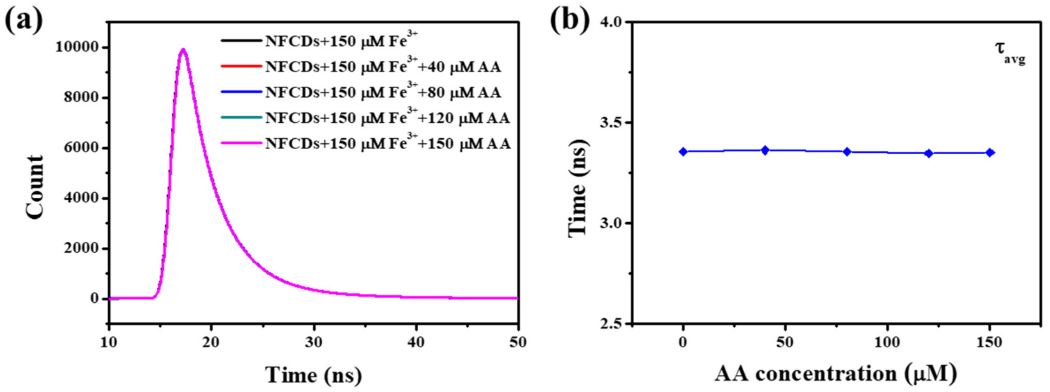

Figure S2. (**a**,**b**) Fluorescence decay of NFCDs with different concentrations of AA added to the NFCDs (0.1 mg mL^{-1}) and Fe^{3+} solution (150 μM).

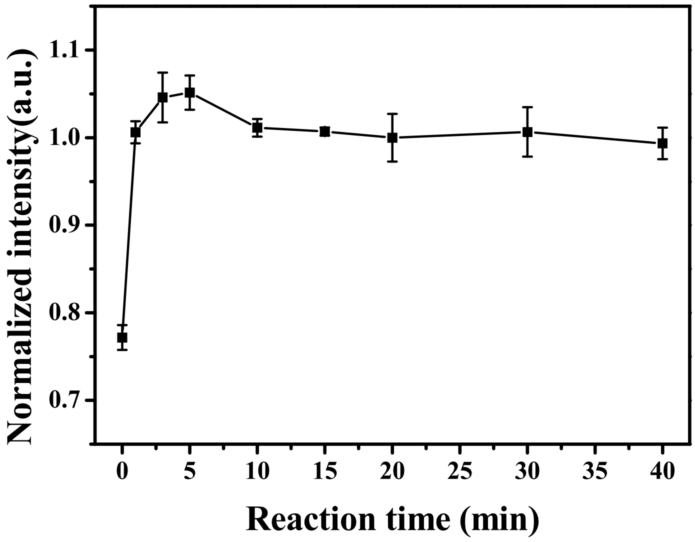

Figure S3. After putting NFCDs into the reaction system of Fe^{3+} (1 mM) and AA (1 mM), the change of its fluorescence intensity over time.

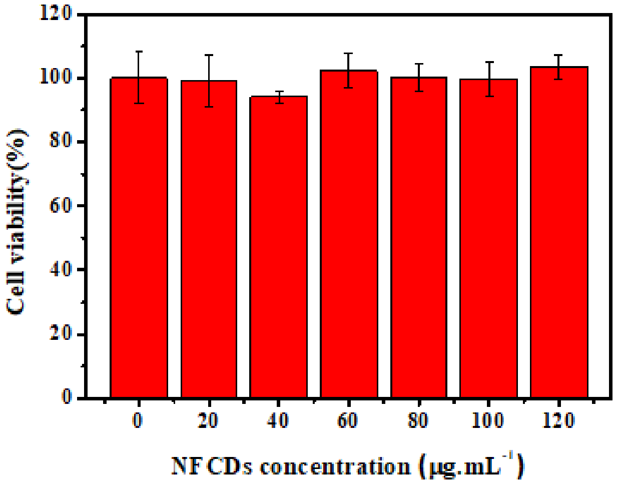

Figure S4. Cell viability in different NFCDs concentration (μg•mL^{-1}).

Table S1. Double exponential fitting fluorescence lifetime of NFCDs at different Fe^{3+} concentrations.

Component / Conce-nitration (μM)	τ_1 (ns)	Amplitude of τ_1	τ_2 (ns)	Amplitude of τ_2	τ_{Avg1} (ns)
0	2.94	4.52	6.77	0.51	3.33
50	3.16	4.76	5.45	0.52	3.38
100	2.98	4.24	5.88	0.57	3.328
150	3.12	4.37	5.12	0.65	3.37

Note: τ_1 and τ_2: fluorescent lifetimes from component 1 and 2, τ_{Avg1}: average fluorescence lifetime; Amplitude of τ_1: rate constant of component 1, Amplitude of τ_2: rate constant of component 2.

Table S2. Double exponential fitting fluorescence lifetime of Fe^{3+}/NFCDs at different AA concentrations

Component / Concent-Ration(μM)	τ_3(ns)	Amplitude of τ_3	τ_4(ns)	Amplitude of τ_4	τ_{Avg2} (ns)
0	2.97	4.72	6.11	0.61	3.33
40	2.89	4.1	5.49	0.72	3.28
80	2.92	4.18	5.9	0.57	3.28
120	2.79	4.04	5.8	0.75	3.26

Note: τ_3 and τ_4: fluorescent lifetimes from component 1 and 2, τ_{Avg2}: average fluorescence lifetime; Amplitude of τ_3: rate constant of component 1, Amplitude of τ_4: rate constant of component 2.
